# Identification of Master Regulators Driving Disease Progression, Relapse, and Drug Resistance in Lung Adenocarcinoma

**DOI:** 10.3389/fbinf.2022.813960

**Published:** 2022-01-28

**Authors:** Qiong Xu, Qiongfang Cha, Hui Qin, Bin Liu, Xueling Wu, Jiantao Shi

**Affiliations:** ^1^ Department of Respiratory Disease, Renji Hospital, Shanghai Jiao Tong University School of Medicine, Shanghai, China; ^2^ State Key Laboratory of Molecular Biology, Shanghai Institute of Biochemistry and Cell Biology, Center for Excellence in Molecular Cell Science, Chinese Academy of Sciences, Shanghai, China

**Keywords:** lung adenocarcinoma, relapse, TKI resistance, immunotherapy, master regulator

## Abstract

**Backgrounds:** Lung cancer is the leading cause of cancer related death worldwide. Current treatment strategies primarily involve surgery, chemotherapy, radiotherapy, targeted therapy, and immunotherapy, determined by TNM stages, histologic types, and genetic profiles. Plenty of studies have been trying to identify robust prognostic gene expression signatures. Even for high performance signatures, they usually have few shared genes. This is not totally unexpected, since a prognostic signature is associated with patient survival and may contain no upstream regulators. Identification of master regulators driving disease progression is a vital step to understand underlying molecular mechanisms and develop new treatments.

**Methods:** In this study, we have utilized a robust workflow to identify potential master regulators that drive poor prognosis in patients with lung adenocarcinoma. This workflow takes gene expression signatures that are associated with poor survival of early-stage lung adenocarcinoma, EGFR-TKI resistance, and responses to immune checkpoint inhibitors, respectively, and identifies recurrent master regulators from seven public gene expression datasets by a regulatory network-based approach.

**Results:** We have found that majority of the master regulators driving poor prognosis in early stage LUAD are cell-cycle related according to Gene Ontology annotation. However, they were demonstrated experimentally to promote a spectrum of processes such as tumor cell proliferation, invasion, metastasis, and drug resistance. Master regulators predicted from EGFR-TKI resistance signature and the EMT pathway signature are largely shared, which suggests that EMT pathway functions as a hub and interact with other pathways such as hypoxia, angiogenesis, TNF-α signaling, inflammation, TNF-β signaling, Wnt, and Notch signaling pathways. Master regulators that repress immunotherapy are enriched with MYC targets, E2F targets, oxidative phosphorylation, and mTOR signaling.

**Conclusion:** Our study uncovered possible mechanisms underlying recurrence, resistance to targeted therapy, and immunotherapy. The predicted master regulators may serve as potential therapeutic targets in patients with lung adenocarcinoma.

## Introduction

Lung cancer is the leading cause of cancer-related death worldwide, and over 2.21 million new cases was reported in 2020 (Ferlay et al., 2021). Non–small cell lung cancer (NSCLC) accounts for 85% of all lung cancer cases (Duma et al., 2019). Histologically, NSCLC is further divided into adenocarcinoma (∼40%), squamous cell carcinoma (∼40%), and large cell carcinoma (∼20%). Current treatment strategies for NSCLC primarily involve surgery, chemotherapy, radiotherapy, molecularly targeted therapy, and immunotherapy, determined by TNM stages, histologic types, and genetic profiles. Surgical resection is the treatment of first choice for early-stage (stage I-II) NSCLC patients. Patients with stage I or II lung adenocarcinoma (LUAD) have 60–70% 5-year survival after surgical resection (Booth et al., 2010). However, more than half of these patients eventually die of recurrences. Even for stage I patients, 11%–48% will relapse in 5 years (Vansteenkiste et al., 2014). With the advent of genomic medicine, EGFR-positive lung cancer was found to represent about 13–47% of LUAD patients (Zhou and Christiani, 2011). For inoperable NSCLC harboring EGFR mutations, EGFR tyrosine kinase inhibitors (TKI) was received as the first-line therapy, but most patients eventually become resistant within 8–14 months (Yu et al., 2013). For inoperable NSCLC without EGFR mutation, PD-1/PD-L1 immune checkpoint inhibitor (ICI) has become the first-line therapy. Immunohistochemistry of PD-L1 protein has emerged as a biomarker for ICI treatment. However, patients with low PD-L1 expressing even undetectable PD-L1 expressing could also benefit from ICI treatment; and patients with high PD-L1 expressing could fail to respond to ICI treatment (Ilie et al., 2016). In summary, although targeted therapy and particularly immunotherapy have revolutionized the treatment landscape of NSCLC, it remains the leading cause of cancer death. Understanding the prognostic factors and underlying molecular mechanisms of NSCLC are vital steps towards new treatments.

Prognostic factors in NSCLC include the stage of diagnosis, histology, smoking status, and gene expression profiles. Plenty of studies have been trying to identify robust prognostic gene expression signatures. However, half of the reported NSCLC prognostic signatures behave just like random gene sets (Tang et al., 2017). The lack of consistent prognostic biomarkers hinders its clinical application. Even for high-performance signatures, they usually have few shared genes. For example, expression signature of six genes including UBE2C, TPX2, MCM2, MCM6, FEN1, and SFN were associated with prognosis in stage I NSCLC patients (Kadara et al., 2009), but in another research, a totally different 15-gene signature was reported as prognostic markers in early-stage NSCLC patients (Zhu et al., 2010). Since a prognostic signature is associated with patient survival and may contain no upstream regulators, which reveal the mechanism of disease progression and may serve as novel therapeutic targets to improve patient survival (Manjang et al., 2021). Regarding EGFR-TKI resistance, the mechanism is relatively well understood (Westover et al., 2018). Genetic alterations account for 90% of the EGFR-TKI resistant cases including EGFR T790M mutation, KRAS mutation, MET amplification, and HER2 amplification (Westover et al., 2018). Epithelial-mesenchymal transition (EMT) (Weng et al., 2019), hypoxia (Lu et al., 2018), and small cell transforming (Leonetti et al., 2021) were known to be linked to acquired EGFR-TKI resistance. Mining key regulators that mediate EGFR-TKI resistance meet the need for new therapeutic strategies. Cancer immunotherapy brings hope to eventually cure cancer by artificial stimulation of the immune system. ICI represents one of the most prominent treatment strategies. Responses to ICI therapy are not just dependent on PD-L1 protein expression and tumor mutation burden (TMB) (Chan et al., 2019), but also affected by T cell infiltration (Soares et al., 2015), T cell dysfunction, and neoantigen load (Lauss et al., 2017). Further identification of key regulators of ICI responses is essential to develop new targets that enhance efficacy.

In this study, we have built a customized workflow to identify potential master regulators that drive poor prognosis of patients with LUAD, based on well-established network inference package ARACNe (Lachmann et al., 2016) and regulator discovery method VIPER (Alvarez et al., 2016). This workflow takes gene expression signatures that are associated with poor survival of early-stage LUAD, EGFR-TKI resistance, and responses to immune checkpoint inhibitors, respectively, and identifies potential master regulators by a regulatory network-based approach. Our study uncovered possible mechanisms underlying recurrence, resistance to EGFR-TKI and ICI, and identified promising therapeutic targets in patients with LUAD.

## Materials and Methods

### Prognostic Signatures for Lung Adenocarcinoma

To evaluate the degree of overlap among published LUAD prognostic gene signatures, we have tested 29 signatures curated in a previous study (Tang et al., 2017) (Supplementary Table S1). EMT signature was defined as the overlaps of differentially expressed genes in two datasets. T cell abundance is estimated by the mean expression of CD8A, CD8B, GZMA, and GZMB (Rooney et al., 2015). The signature of T cell dysfunction is taken from a published study (Jiang et al., 2018). GSE123066 and GSE121634, which were available from NCBI GEO (https://www.ncbi.nlm.nih.gov/geo/), were used to define a TKI resistant signature. In dataset GSE123066, EGFR TKI resistant samples were compared to parental cell lines, and differentially expressed genes were identified using limma (version 3.48.1) with default parameters (Ritchie et al., 2015). For dataset GSE121634, differentially expressed genes were identified using DESeq2 (version 1.32.0) (Love et al., 2014) with default parameters. The EGFR TKI signature was defined as the genes that were significantly upregulated in both datasets (FDR < 5%).

### Gene Expression Datasets

All gene expression datasets used in this study were publicly available. Seven lung adenocarcinoma datasets, including GSE14814 (Zhu et al., 2010), GSE37745 (Botling et al., 2013), GSE50081 (Der et al., 2014), GSE68465 (Director’s Challenge Consortium for the Molecular Classification of Lung Adenocarcinomo et al., 2008), GSE19188 (Hou et al., 2010), GSE31210 (Yamauchi et al., 2012), and TCGA-LUAD (Cancer Genome Atlas Research Network, 2014) were used to construct transcriptional regulatory networks (Supplementary Table S2). Except for TCGA-LUAD, samples on all other datasets were profiled with Affymetrix gene expression arrays and pre-processed with Bioconductor Affy package using the default parameters.

### Identification of Master Regulators

For each LUAD dataset with a reasonablely large sample size (*n* > 100), the transcriptional regulatory network (TRN) was constructed using ARACNe-AP (Lachmann et al., 2016) with default parameters (MI *p*-value = 10^–8^, 100 bootstraps). The TRN for TCGA-LUAD was extracted from the Bioconductor data package aracne. networks (version 1.18.0). For a given prognostic signature, patient samples were stratified by the enrichment of this signature as quantified by Wilcoxon rank-sum test. The top 50 samples with the highest enrichment scores were compared to those with the lowest by VIPER (version 1.26.0) (Alvarez et al., 2016) to identify potential master regulators with a *p*-value cutoff of 0.01. With this workflow, each signature was tested on seven independent LUAD datasets.

### Evaluation of the Workflow

For a robust workflow, statistically equivalent signatures result in highly concordant master regulators. To evaluate the robustness of the workflow, we have compared two well-established LUAD prognostic signatures. One is the top-scoring signature (Xie signature) (Xie et al., 2011) described in a review study (Tang et al., 2017), the other one is embryonic stem cell (ESC) signature (Ben-Porath et al., 2008). Jacard index was used to measure similarities between master regulators identified from two signatures. To capture dynamic ranges, 80% of genes were sampled without replacement from two signatures, respectively, the resulting master regulators were then compared using Jacard index. This procedure was repeated 100 times, and the median Jacard index was reported. Similarly, random signatures sampled from the background were compared 100 times.

### Pathway and Survival Analysis

Hypergeometric test was used to evaluate the enrichment of pathways in a list of genes. Hallmark and Gene Ontology (GO) gene sets curated by MSigDB (version 5.0) (Subramanian et al., 2005; Liberzon et al., 2011; Liberzon et al., 2015) were used in this study. Given two groups of samples, the survival difference was compared with the Kaplan-Meier method, implemented in R package survival (version 3.2).

### Code and Data Availability

The code and data are publicly available at Github (https://github.com/JiantaoShi/LungMR).

## Results

### A Robust Workflow to Identify Master Regulators

**FIGURE 1:**
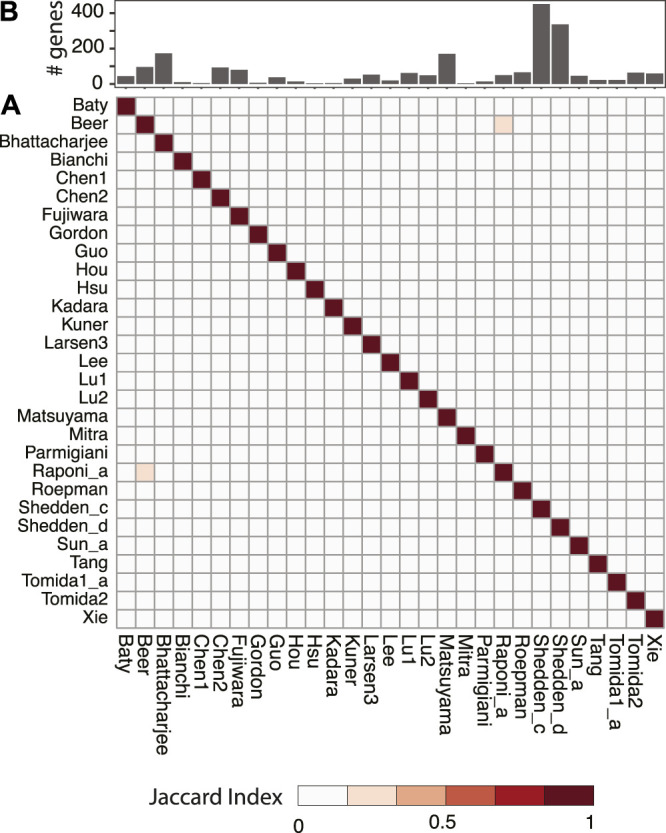
Pairwise comparison of published prognostic gene signatures for LUAD. All signatures were taken from a review study (Tang et al., 2017) and the original references were listed in Supplementary Table S1. Jaccard index was used to quantify the level of overlap between two signatures and shown as a heatmap **(A)**. The number of genes in each signature was shown as a bar plot **(B)**.

**FIGURE 2:**
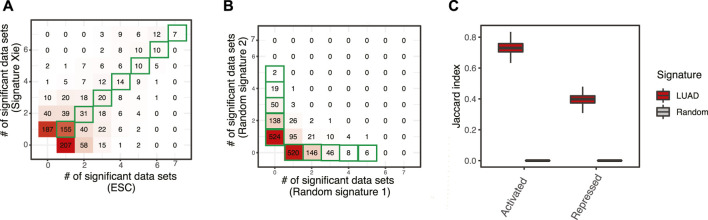
A workflow to identify master regulators driving prognosis in patients with LUAD. **(A)** Comparison of master regulators predicted with two well-established LUAD signatures, one was the best-scoring signature benchmarked in a previous review study (Xie signature) (Xie et al., 2011), the other is embryonic stem cells signature (ESC) (Ben-Porath et al., 2008). Both signatures were tested across seven LUAD gene expression datasets and master regulators were identified with a *p*-value cutoff of 0.01 (Section 2). The results were summarized to show how many times a master regulator was identified and represented as a 2D histogram. **(B)** Comparison of master regulators predicted with random signatures. **(C)** Evaluation of robustness. The Jacard index shows the similarity of master regulators predicted from subsampling of LUAD signatures (100 times), as well as that from random background.

**FIGURE 3:**
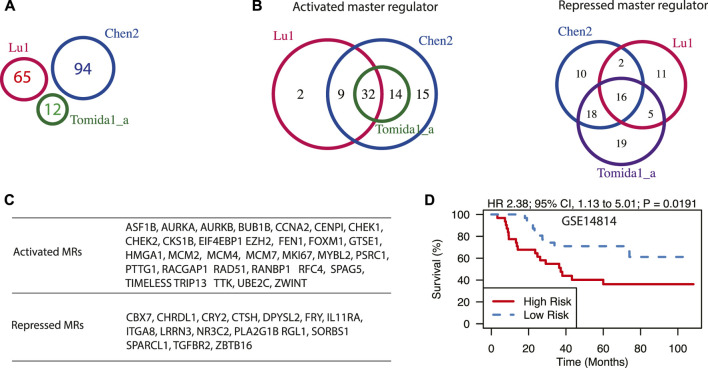
Master regulators driving prognosis in early-stage LUAD. **(A)** A Venn diagram of three prognostic signatures for early-stage LUAD. Signature names as well as the number of genes in each signature were shown. **(B)** The Venn diagram of activated and repressed master regulators, predicted from three prognostic signatures for early-stage LUAD. **(C)** The lists of shared master regulators that are predicted from all three signatures. **(D)** Expression of shared master regulators is associated with poor survival in patients with stage I LUAD in dataset GSE14814. Hazard ratio (HR) and log-rank test *p*-value are also shown.

Numerous studies have been trying to identify prognostic signatures for LUAD. Though a significant proportion of them perform better than random gene sets, few genes are shared. We have evaluated 29 lung adenocarcinoma signatures curated in a previous study (Supplementary Table S1) (Tang et al., 2017) and found only two pairs of signatures shared more than 10% of their genes, as measured by Jaccard index (Figure 1). This is not totally unexpected, since genes are correlated, and different studies may identify combinations of different genes. We have built a workflow to identify master regulators that potentially drive poor survival in patients with LUAD (Section 2, Supplementary Figure S1). To demonstrate the effectiveness of this method, we have tested the best-scoring signature (Xie signature) (Xie et al., 2011), benchmarked in a previous review study (Tang et al., 2017). Since it is associated with overall survival, we have compared it to the embryonic stem cell (ESC) signature which is known to be associated with poor survival in multiple cancer types including LUAD (Ben-Porath et al., 2008). The master regulators predicted with these two signatures are highly concordant, as demonstrated by the two-dimensional histogram (Figure 2A). For instance, seven master regulators, including BUB1B, ECT2, MCM6, RACGAP1, TOP2A, WDR12, and ZWINT, were identified by both signatures across all seven datasets tested. In contrast, master regulators predicted from two random signatures have little overlap. None of the genes that were identified in four or more datasets were shared (Figure 2B). To further evaluate the robustness of this workflow, we compared the overlap between results identified from subsampled Xie signature and ESC signature (Section 2). For LUAD signatures, the Jacard index reaches 0.7297 and 0.3988 for activated and repressed master regulators, respectively; in contrast, a value of 0 was observed for random signatures (Figure 3C).

### Master Regulators Driving Poor Prognosis in Patients With Early-Stage Lung Adenocarcinoma

Using the workflow described above, we first explored the master regulators driving relapses in lung cancer patients in early stages (stage I/II). We have chosen three gene expression signatures from the top five best-scoring ones according to a previous review study (Tang et al., 2017). They are denoted as Lu et al. (2006), Chen et al. (2011), and Tomida et al. (2004). Interestingly, though these three gene signatures have no shared genes (Figure 3A), the predicted master regulators were largely shared (Figure 3B). For instance, 46 activated master regulators were identified with signature Tomida et al. (2004), and all of them were included in results from signature Chen2. Similarly, 41 out of 43 activated master regulators identified from signature Lu were also identified by signature Chen2. When overlapping results from all three signatures, 32 activated and 16 repressed master regulators were identified (Figure 3C). Many of them have been reported to be associated with the survival of patients with LUAD. FOXM1 was demonstrated to be necessary and sufficient to cause the progression of lung adenomas into invasive mucinous adenocarcinomas *in vivo* by activating AGR2 (Milewski et al., 2017). It was also shown to promote LUAD invasion and metastasis by upregulating SNAIL (Wei et al., 2015). EIF4EBP1 (Seki et al., 2010) and HMGA1 (Qiao et al., 2021) were shown to be potential markers for predicting recurrence and poor prognosis in stage I LUAD. The activated master regulators are predominantly enriched with GO term Cell Cycle (*p*-value < 10^–16^, Supplementary Table S3), and they generally fall into three functional categories based on previous studies: cell proliferation, cell metastasis, and drug resistance. Cell cycle-related genes such as AURKA, AURKB, GTSE1, CCNA2, and MYBL2 were shown to promote tumor progression of LUAD (Musa et al., 2017; Ruan et al., 2017; Galetta and Cortes-Dericks, 2020; Zhang et al., 2021). Some of the predicted master regulators, including PTTG1 (Li et al., 2013), RFC4 (Liu et al., 2021), TRIP13 (Zhang et al., 2019), BUB1B (Chen et al., 2015), TTK (Tsai et al., 2020), and ZWINT (Peng et al., 2019) are capable of promoting migration, invasion, and metastasis of lung cancer cells. CHEK1 (Bartucci et al., 2012), FEN1 (He et al., 2017), and UBE2C (Wu et al., 2019) were shown to confer chemotherapy resistance in NSCLC. Chemotherapeutic resistance is usually associated with overactive HR repair mechanisms, which could be driven by overexpression of RAD51 (Ward et al., 2015). Furthermore, some of the predicted master regulators have been tested as potential drug targets. For example, EZH2 is activated in many cancer types including LUAD and may serve as an opportunity for targeted therapy in lung cancer (Zhang et al., 2016). The expression of predicted master regulators is significantly associated with poor survival in early-stage LUAD in dataset GSE14814 (*p* = 0.0191, Hazard ratio = 2.38) (Figure 3D).

### Master Regulators Driving EGFR-TKI Resistance

**FIGURE 4:**
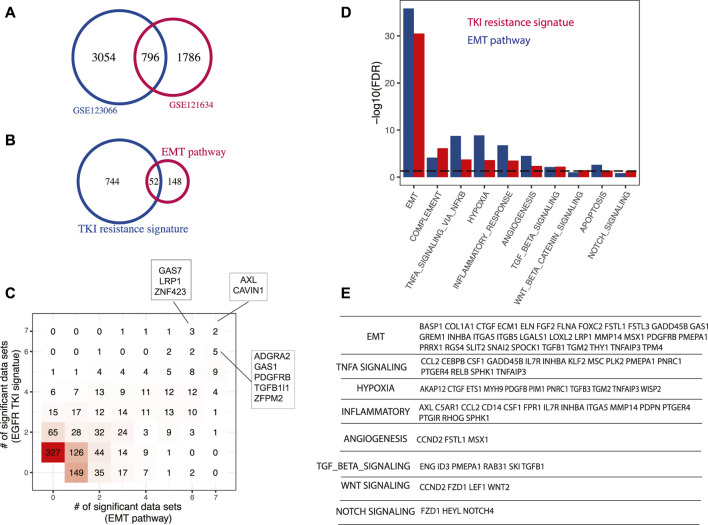
Master regulators driving EGFR-TKI resistance. **(A)** A new EGR-TKI resistance signature. EGFR-TKI resistant samples were compared to parental cell lines and the signature was defined as the genes that were significantly upregulated in two independent datasets. **(B)** Enrichment of EMT pathway in EGFR-TKI resistance signature. **(C)** Comparison of master regulators predicted with EGFR-TKI signature and EMT pathway signature. Both signatures were tested across seven LUAD gene expression datasets and master regulators were identified with a *p*-value cutoff of 0.01 (Section 2). The results were summarized to show how many times a master regulator was identified and represented as a 2D histogram. **(D)** Pathways enriched in top-ranking master regulators predicted using TKI resistance signature or EMT pathway signature. **(E)** Selected master regulators grouped by enriched pathways. To avoid redundancy, a gene in multiple pathways was only assigned to the one with higher significance.

We performed differential gene expression between EGFR-TKI resistant and parental EGFR mutant cell lines using two public datasets GSE123066 (Becker et al., 2019) and GSE121634 (Nilsson et al., 2020), and defined a new signature as the shared genes (Figure 4A). This 796-gene signature is significantly enriched with EMT signaling pathway (hypergeometric test *p*-value < 10^–16^) (Figure 4B), which is an extensively validated pathway involved in EGFR-TKI resistance. The master regulators identified with this signature and the EMT pathway signature were largely shared, as indicated by the 2D histogram (Figure 4C). AXL and CAVIN1 were identified by both signatures across all seven datasets tested. AXL is one of the best-known genes involved in TKI resistance (Zhang et al., 2012) and it was demonstrated to confers intrinsic resistance to Osimertinib, a third-generation EGFR TKI that was approved for the treatment of EGFR-T790M-positive NSCLC (Taniguchi et al., 2019). At the pathway level, besides EMT signaling pathway, several known pathways have been recovered (FDR < 5%) (Figure 4D). For instance, hypoxia was shown to promote resistance to EGFR inhibition in NSCLC cells *via* the histone demethylases (Lu et al., 2018). However, the master regulators that mediate the effect of hypoxia in lung cancer remain unelucidated. Our prediction was validated by studies of these master regulators in other biological systems. AKAP12 (Finger et al., 2015), CTGF (Higgins et al., 2004), ETS1 (Qiao et al., 2015), PDGFB (Schito et al., 2012), PIM1 (Casillas et al., 2018), TGFB3 (Schaffer et al., 2003), TGM2 (Jin et al., 2012), and WISP2 (Fuady et al., 2014) were shown to be essential to mediate hypoxia effect in various cell systems (Figure 4E). Angiogenesis pathway was also enriched in top predicted master regulators and it was known to be an important prognostic factor and therapeutic target in LUAD. Targeting VEGFR2, the VEGFA receptor that is essential for endothelial cell functions associated with angiogenesis, enhances the anti-tumor activity of EGFR-TKIs in NSCLC with EGFR-TKI resistance (Li et al., 2017). Angiogenesis-related master regulators identified in this study such as CCND2, FSTL1, and MSX1 represent new options to module angiogenesis in LUAD. Consistent with previous studies, our results show that master regulators in TNF-α signaling pathway mediate resistance to EGFR inhibition in LUAD (Gong et al., 2018). TGF-β signal pathway is also identified as a mechanism of TKI resistance, which has been supported by several previous studies (Serizawa et al., 2013). Cancer cells can increase their production of active TGF-β during the development of EGFR-TKI resistance, which triggers EMT and allow the cells to become invasive (Jakobsen et al., 2016). Interestingly, a group of inflammation master regulators was predicted to drive EGFR-TKI resistance, including AXL which is one of the well-known genes involved in TKI resistance, as described above (Zhang et al., 2012). ITGA5 was known to promote cancer cell migration and invasion through the FAK/STAT3/AKT signaling pathway in TKI-resistant NSCLC (Yang et al., 2021). CCL2, a potent chemokine for macrophages and a variety of other immune cells, has been reported to play an indispensable role in the process of TKI resistance (Xiao et al., 2020). Besides, studies have shown that Notch signaling leads to acquired resistance to EGFR-TKI and Notch inhibition overcomes resistance in LUAD (Bousquet Mur et al., 2020). And there is crosstalk between Notch and Wnt signaling pathways in EGFR mutant NSCLC (Arasada et al., 2018).

### Master Regulators Driving Response in Immunotherapy

**FIGURE 5:**
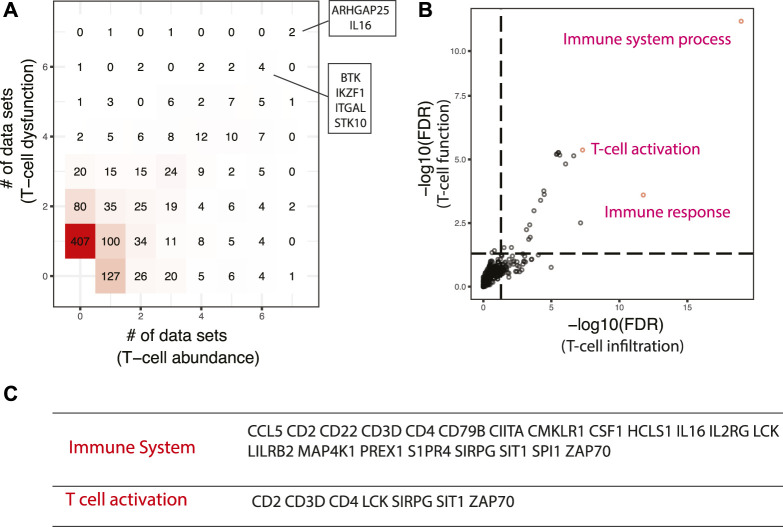
Master regulators that are associated with enhanced responses in immune checkpoint blockade-based immunotherapy. **(A)** Comparison of master regulators predicted from T cell infiltration and T cell dysfunction signatures. The same set of master regulators that were identified in at least six datasets were shown. **(B)** Pathways enriched in top-ranking master regulators. The top three most enriched pathways were shown. **(C)** Selected master regulators grouped by enriched pathways.

Two signatures were used to identify master regulators promoting immunotherapy that is based on immune checkpoint blockade, one quantifying the abundance of tumor-infiltrating T cells (Rooney et al., 2015), and the other characterizing T cell dysfunction (Jiang et al., 2018). Activated master regulators are correlated with higher T cell abundance and enhanced T cell activity. Six activated master regulators have been predicted to regulate both signatures across six or more datasets (Figure 5A), including ARHGAP25, IL16, BTK, IKZF1, ITGAL, and STK10, most of which were reported to function in immune-related pathways. IL16 is a chemoattractant for CD4^+^ cells and a modulator of T cell activation (Mathy et al., 2000). BTK is known to be essential for BCR-mediated proliferation and survival. A recent study showed that BTK can promote T cell activation by phosphorylation of PLCγ1 upon TCR engagement (Xia et al., 2020). IKZF1 is a lymphocyte-specific transcription factor that controls a wide spectrum of immune cell development, especially CD4^+^ T cell subsets (Wu et al., 2006). Intriguingly, IKZF1 was demonstrated to enhance immune infiltrate recruitment in several cancer types including LUAD, and thus susceptibility to immunotherapy (Chen et al., 2018). ITGAL is a pan-leukocyte marker and is involved in a variety of immune phenomena including T cell-mediated killing. Though its function in cancer has not been reported, it was shown to play an essential role in cytotoxic T Cell accumulation and activation in adipose tissue (Jiang et al., 2014). At the pathway level, immune system and T cell activation pathways are significantly enriched in the top-ranking master regulators (Figures 5B,C).

**FIGURE 6:**
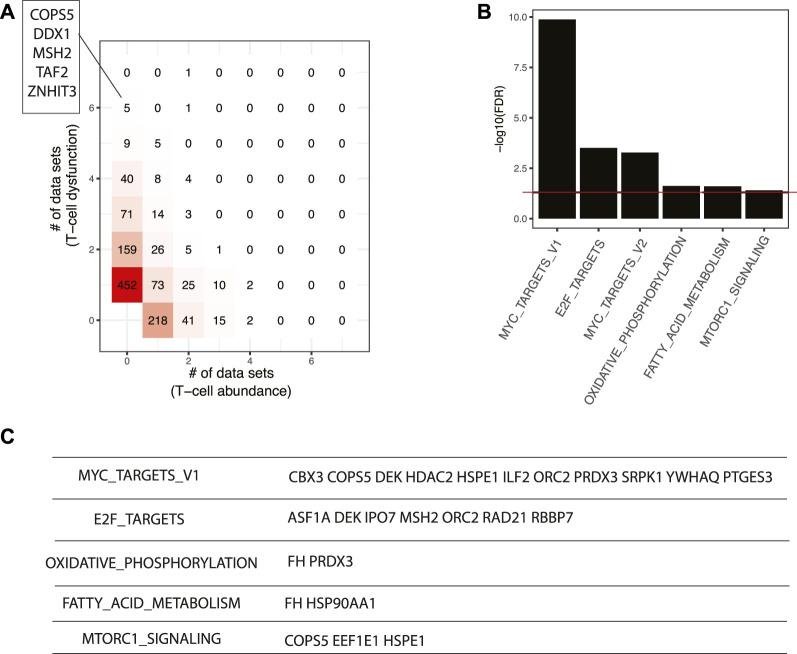
Master regulators that are associated with poor responses in ICB-based immunotherapy. **(A)** Comparison of master regulators predicted from T cell infiltration and T cell dysfunction signatures. The master regulators predicted in at least six datasets with T cell dysfunction signature were shown. **(B)** Pathways enriched in top-ranking master regulators. **(C)** Selected master regulators grouped by enriched pathways.

Master regulators that result in lower T cell abundance and T cell dysfunction are more interesting, since they could be repressed by inhibitors to enhance cancer immunotherapy. Seven master regulators, including COPS5, DDX1, GGCT, MSH2, TAF2, TFB2M, and ZNHIT3, were identified in at least six datasets with the signature of T cell dysfunction (Figure 6A). COPS5 is required for TNF-α-mediated PD-L1 stabilization in breast cancer cells and inhibition of COPS5 sensitized cancer cells to anti-CTLA4 therapy (Lim et al., 2016). MSH2 is a known DNA mismatch repair gene and mismatch repair deficiency is a predictor of immune response for anti-PD-1/PD-L1 immunotherapy efficacy (Zhao et al., 2019). At the pathway level, we found top-ranking master regulators were enriched with MYC targets, E2F targets, oxidative phosphorylation, fatty acid metabolism, and mTORC1 signaling (FDR < 5%) (Figure 6B). It has been shown that MYC amplified tumors are associated with suppressed immune cell infiltrates in neuroblastoma and melanoma models (Wu et al., 2021). CBX3 is a putative target of MYC (Figure 6C) and may serve as a new diagnostic biomarker and a potential target for immunotherapy in gastric cancer (Lin et al., 2020). E2F targets are essential regulators of the cell cycle which is a classical therapeutic target in cancer. ASF1A is one of E2F targets predicted to repress cancer immunity and was identified as a critical regulator of sensitivity to anti–PD-1 therapy in LUAD (Li et al., 2020). Furthermore, researchers found that pharmacological inhibitors of cyclin-dependent kinase 4 (CDK4) and CDK6 boost tumor immunogenicity (Goel et al., 2017). The mTOR pathway regulates cancer cell proliferation and tumor angiogenesis. Clinically, mTOR inhibitor only shows modest anticancer efficacy due to resistance and immunosuppressive properties (El Hage and Dormond, 2021), which suggests that mTOR inhibitors represent a therapeutic opportunity to promote the efficacy of cancer immunotherapy (Esfahani et al., 2019). Given the support from previous studies in other cancer systems, it is worth further testing the pathways and master regulators predicted by our workflow with comprehensive experiments.

## Discussion

In this study, we have systemically identified master regulators driving prognosis in patients with LUAD, treated with surgical resection, EGFR-TKI, and ICB-based cancer immunotherapy. This network-based workflow is highly robust, and it identified concordant master regulators from prognostic signatures with no shared genes. This is an important and unique feature of this workflow, as signatures tend to be divergent but upstream regulators can be convergent. Furthermore, running this workflow requires only gene expression data and predefined signatures, which make it useful for a variety of biological systems.

With this workflow, we have found that the majority of the master regulator driving poor prognosis in early-stage LUAD are cell-cycle related according to Gene Ontology annotation. However, they were demonstrated experimentally to promote a spectrum of processes such as tumor cell proliferation (Musa et al., 2017; Ruan et al., 2017; Galetta and Cortes-Dericks, 2020; Zhang et al., 2021), invasion (Li et al., 2013), metastasis (Liu et al., 2021), and drug resistance (Bartucci et al., 2012; He et al., 2017; Wu et al., 2019). This analysis not only uncovered the mechanism of regulation, but also identified novel molecular targets for treating LUAD. For instance, RAD51 and SPAG5 were identified to be associated with poor survival in patients with early stage LUAD but their function in lung cancer has not been extensively characterized (Qiao et al., 2005; Huang and Li, 2020). Studies in other cancer types suggest they are promising targets to follow up. SPAG5 is a mitotic spindle protein, which promotes cancer cell proliferation and invasion by activating AKT/mTOR pathway in bladder and hepatocellular cancer (Liu et al., 2018; Yang et al., 2018). Knocking down SPAG5 increased the S-phase cell population and decreased the expression of c-MYC target genes, including the DNA repair proteins RAD51 and BRCA1/2 in triple-negative breast cancer (Li et al., 2019). In NSCLC, miR-1179 suppresses cancer cell growth and invasion by direct targeting SPAG5 (Song et al., 2018). Furthermore, SPAG5 was shown to confer resistance to cisplatin-induced apoptosis in bladder urothelial carcinoma cells (Liu et al., 2018). Taken together, SPAG5 represents one of the novel therapeutic targets that promote poor prognosis in LUAD.

Given the significance of targeted therapy in lung cancer, the mechanism of EGFR-TKI resistance was extensively characterized and mainly focused on EMT pathway (Westover et al., 2018; Weng et al., 2019). It is noted that master regulators predicted from EGFR-TKI resistance signature and EMT pathway signature are largely shared, which suggests that EMT pathway functions as a hub and interact with other pathways such as hypoxia, angiogenesis, TNF-α signaling, inflammation, TNF-β signaling, Wnt, and Notch signaling pathways. Our integrative analysis not only uncovers key pathways that mediate EGFR-TIK resistance but also suggests new options to modulate these pathways to overcome resistance. Some master regulators have been characterized in NSCLC (Zhang et al., 2012; Yang et al., 2021), and some have been reported in other cancer types, which pave the way for successful validation in lung cancer. For instance, FOXC2 is an EMT regulator (Thiery et al., 2009) and promotes cell migration and invasion through EMT in breast cancer, ovarian cancer, prostate cancer, and lung cancer (Hollier et al., 2013). It also confers chemoresistance in multiple cancer types, including nasopharyngeal carcinomas (Zhou et al., 2015), breast cancer (Cai et al., 2015), and lung cancer. It is a promising therapeutic target to overcome EMT-mediated EGFR-TKI resistance in LUAD.

With cancer immune signatures, we have identified master regulators potentially enhance or suppress ICB-based immunotherapy response in LUAD. ICB was designed to help the immune system recognize and attack cancer cells (Jiang et al., 2018). In concordant with this concept, master regulators that activate the immune system especially T cells are capable of enhancing immune response. Master regulators that repress immunotherapy are enriched with MYC targets, E2F targets, oxidative phosphorylation, and mTOR signaling. The emerging concept of metabolic modulation of immunity is opening a new window for cancer immunotherapy (Guerra et al., 2020). Our prediction suggests that oxidative phosphorylation is a metabolic target for lung cancer immunotherapy, which has been demonstrated in melanoma (Najjar et al., 2019). Several genes in these pathways were demonstrated to be involved in immunotherapy response, such as COPS5 (Lim et al., 2016), MSH2 (Zhao et al., 2019), CBX3 (Lin et al., 2020), ASF1A (Li et al., 2020) and HSP90A (Song et al., 2020). Some of the predicted master regulators are known to function in cancer cells, but their role in immunotherapy response is worth further exploring. For example, SRPK1, an enzyme that phosphorylates splicing factors rich in serine/arginine domains (SR proteins), is associated with poor survival in various cancers (Patel et al., 2019). In lung cancer, SRPK1 inhibition suppresses angiogenesis, metastasis, and the acquisition of a cancer stem cell phenotype (Wu et al., 2017; Wagner et al., 2019). SRPK1 is associated with lung cancer progression by activating the transcriptional activity of the beta-catenin/T-cell factor (TCF) complex (Liu et al., 2016). Our prediction suggests that SRPK1 represses immunotherapy response through the MYC pathway.

The workflow described in this study not only identified well-characterized master regulators, but also discovered novel ones that can be potentially tested as therapeutic targets in LUAD. We believe it can be used on other types of lung cancers such as lung squamous cell cancers and small cell lung cancer.

## Data Availability

Publicly available datasets were analyzed in this study. The code and data are publicly available at Github (https://github.com/JiantaoShi/LungMR).

## Supplementary Material

Supplementary Figure S1 The workflow diagram. Regulons which were pre-computed from 7 LUAD gene expression datasets are ready to use. For a given prognostic signature, master regulators were identified with two analytical steps. Tools and methods used in this workflow were highlighted in green.
