# Supplementary figures and images for: Identification of Master Regulators Driving Disease Progression, Relapse, and Drug Resistance in Lung Adenocarcinoma
Source: Front Bioinform. 2022 Jan 28;2:813960. doi: 10.3389/fbinf.2022.813960 (PMC9580914; doi:10.3389/fbinf.2022.813960)

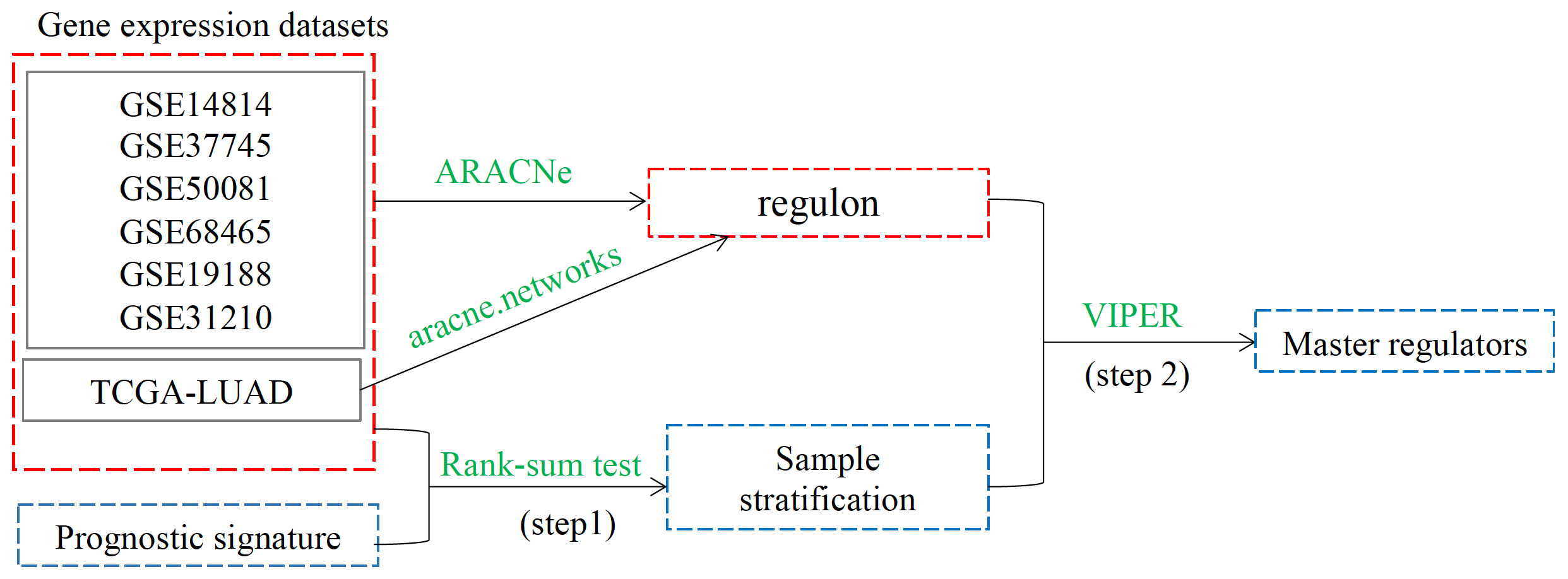

Supplement: Supplementary file 1 [file Image1.TIFF]
