# Supplementary material for: Identification of Master Regulators Driving Disease Progression, Relapse, and Drug Resistance in Lung Adenocarcinoma
Source: Front Bioinform. 2022 Jan 28;2:813960. doi: 10.3389/fbinf.2022.813960 (PMC9580914; doi:10.3389/fbinf.2022.813960)
Supplement: Supplementary file 2 [file Table1.DOCX]

**Supplementary Table 1.** Sources of gene expression signatures curated from public studies and compared in Figure 1.

| **Signature ID** | **Histology** | **Total Probes** | **Citation** |
| --- | --- | --- | --- |
| **Baty** | NSCLC | 44 | [1] |
| **Beer** | ADC | 100 | [2] |
| **Bhattacharjee** | ADC | 175 | [3] |
| **Bianchi** | ADC | 10 | [4] |
| **Chen1** | NSCLC | 5 | [5] |
| **Chen2** | other | 102 | [6] |
| **Fujiwara** | NSCLC | 100 | [7] |
| **Gordon** | ADC | 7 | [8] |
| **Guo** | ADC | 38 | [9] |
| **Hou** | NSCLC | 17 | [10] |
| **Hsu** | NSCLC | 4 | [11] |
| **Kadara** | ADC | 5 | [12] |
| **Kuner** | ADC,SCC | 30 | [8] |
| **Larsen3** | ADC | 54 | [13] |
| **Lee** | NSCLC | 20 | [14] |
| **Lu1** | ADC,SCC | 64 | [15] |
| **Lu2** | NSCLC | 51 | [16] |
| **Matsuyama** | NSCLC | 201 | [17, 18] |
| **Mitra** | NSCLC | 4 | [19] |
| **Parmigiani** | Lung Cancer | 14 | [20] |
| **Raponi_a** | ADC | 50 | [21] |
| **Roepman** | NSCLC | 72 | [22] |
| **Shedden_c** | ADC | 459 | [23] |
| **Shedden_d** | ADC | 348 | [23] |
| **Sun_a** | ADC | 50 | [24] |
| **Tang** | ADC | 23 | [25] |
| **Tomida1_a** | NSCLC | 25 | [26] |
| **Tomida2** | ADC | 82 | [27] |
| **Xie** | NSCLC | 59 | [28] |

**Supplementary Table 2.** LUAD datasets used for for identification of master regulators.

| **Data Set** | **# Samples** | **Ref.** |
| --- | --- | --- |
| **GSE14814** | 133 | [29] |
| **GSE37745** | 106 | [30] |
| **GSE50081** | 181 | [31] |
| **GSE68465** | 462 | [32] |
| **GSE19188** | 156 | [33] |
| **GSE31210** | 246 | [34] |
| **TCGA-LUAD** | 515 | [35] |

**Supplementary Table 3.** GO biological process ontology enriched in active master regulators that poor prognosis in early stage LUAD.

| **Gene Set Name** | **Gene set Size (K)** | **# Overlap (k)** | **k/K** | **p-value** | **FDR q-value** |
| --- | --- | --- | --- | --- | --- |
| GOBP_CELL_CYCLE_PROCESS | 1415 | 29 | 0.0205 | 2.21E-39 | 1.65E-35 |
| GOBP_MITOTIC_CELL_CYCLE | 1032 | 26 | 0.0252 | 2.36E-36 | 8.84E-33 |
| GOBP_CELL_CYCLE | 1872 | 29 | 0.0155 | 7.68E-36 | 1.92E-32 |
| GOBP_REGULATION_OF_CELL_CYCLE | 1211 | 25 | 0.0206 | 1.89E-32 | 3.54E-29 |
| GOBP_CHROMOSOME_ORGANIZATION | 1244 | 24 | 0.0193 | 3.72E-30 | 5.57E-27 |
| GOBP_REGULATION_OF_CELL_CYCLE_PROCESS | 789 | 20 | 0.0253 | 9.72E-27 | 1.21E-23 |
| GOBP_CELL_CYCLE_PHASE_TRANSITION | 641 | 19 | 0.0296 | 1.48E-26 | 1.58E-23 |
| GOBP_MITOTIC_NUCLEAR_DIVISION | 296 | 16 | 0.0541 | 2.57E-26 | 2.40E-23 |
| GOBP_ORGANELLE_FISSION | 486 | 17 | 0.035 | 8.70E-25 | 7.23E-22 |
| GOBP_POSITIVE_REGULATION_OF_CELL_CYCLE | 395 | 14 | 0.0354 | 2.40E-20 | 1.80E-17 |

**References**

1. Baty F, Facompre M, Kaiser S, Schumacher M, Pless M, Bubendorf L, Savic S, Marrer E, Budach W, Buess M *et al*: **Gene profiling of clinical routine biopsies and prediction of survival in non-small cell lung cancer**. *Am J Respir Crit Care Med* 2010, **181**(2):181-188.

2. Beer DG, Kardia SLR, Huang CC, Giordano TJ, Levin AM, Misek DE, Lin L, Chen GA, Gharib TG, Thomas DG *et al*: **Gene-expression profiles predict survival of patients with lung adenocarcinoma**. *Nat Med* 2002, **8**(8):816-824.

3. Bhattacharjee A, Richards WG, Staunton J, Li C, Monti S, Vasa P, Ladd C, Beheshti J, Bueno R, Gillette M *et al*: **Classification of human lung carcinomas by mRNA expression profiling reveals distinct adenocarcinoma subclasses**. *Proc Natl Acad Sci U S A* 2001, **98**(24):13790-13795.

4. Bianchi F, Nuciforo P, Vecchi M, Bernard L, Tizzoni L, Marchetti A, Buttitta F, Felicioni L, Nicassio F, Di Fiore PP: **Survival prediction of stage I lung adenocarcinomas by expression of 10 genes**. *The Journal of clinical investigation* 2007, **117**(11):3436-3444.

5. Chen HY, Yu SL, Chen CH, Chang GC, Chen CY, Yuan A, Cheng CL, Wang CH, Terng HJ, Kao SF *et al*: **A five-gene signature and clinical outcome in non-small-cell lung cancer**. *The New England journal of medicine* 2007, **356**(1):11-20.

6. Chen DT, Hsu YL, Fulp WJ, Coppola D, Haura EB, Yeatman TJ, Cress WD: **Prognostic and predictive value of a malignancy-risk gene signature in early-stage non-small cell lung cancer**. *J Natl Cancer Inst* 2011, **103**(24):1859-1870.

7. Fujiwara T, Hiramatsu M, Isagawa T, Ninomiya H, Inamura K, Ishikawa S, Ushijima M, Matsuura M, Jones MH, Shimane M *et al*: **ASCL1-coexpression profiling but not single gene expression profiling defines lung adenocarcinomas of neuroendocrine nature with poor prognosis**. *Lung Cancer* 2012, **75**(1):119-125.

8. Gordon GJ, Richards WG, Sugarbaker DJ, Jaklitsch MT, Bueno R: **A prognostic test for adenocarcinoma of the lung from gene expression profiling data**. *Cancer Epidemiology Biomarkers & Prevention* 2003, **12**(9):905-910.

9. Guo L, Ma Y, Ward R, Castranova V, Shi X, Qian Y: **Constructing molecular classifiers for the accurate prognosis of lung adenocarcinoma**. *Clinical cancer research* 2006, **12**(11):3344-3354.

10. Hou J, Aerts J, den Hamer B, van IJcken W, den Bakker M, Riegman P, van der Leest C, van der Spek P, Foekens JA, Hoogsteden HC: **Gene expression-based classification of non-small cell lung carcinomas and survival prediction**. *Plos One* 2010, **5**(4):e10312.

11. Hsu YC, Yuan S, Chen HY, Yu SL, Liu CH, Hsu PY, Wu G, Lin CH, Chang GC, Li KC *et al*: **A four-gene signature from NCI-60 cell line for survival prediction in non-small cell lung cancer**. *Clin Cancer Res* 2009, **15**(23):7309-7315.

12. Kadara H, Behrens C, Yuan P, Solis L, Liu D, Gu X, Minna JD, Lee JJ, Kim E, Hong WK *et al*: **A five-gene and corresponding protein signature for stage-I lung adenocarcinoma prognosis**. *Clin Cancer Res* 2011, **17**(6):1490-1501.

13. Larsen JE, Pavey SJ, Passmore LH, Bowman RV, Hayward NK, Fong KM: **Gene expression signature predicts recurrence in lung adenocarcinoma**. *Clin Cancer Res* 2007, **13**(10):2946-2954.

14. Lee ES, Son DS, Kim SH, Lee J, Jo J, Han J, Kim H, Lee HJ, Choi HY, Jung Y *et al*: **Prediction of recurrence-free survival in postoperative non-small cell lung cancer patients by using an integrated model of clinical information and gene expression**. *Clin Cancer Res* 2008, **14**(22):7397-7404.

15. Lu Y, Lemon W, Liu PY, Yi Y, Morrison C, Yang P, Sun Z, Szoke J, Gerald WL, Watson M *et al*: **A gene expression signature predicts survival of patients with stage I non-small cell lung cancer**. *PLoS Med* 2006, **3**(12):e467.

16. Lu Y, Wang L, Liu P, Yang P, You M: **Gene-expression signature predicts postoperative recurrence in stage I non-small cell lung cancer patients**. *Plos One* 2012, **7**(1):e30880.

17. Matsuyama Y, Suzuki M, Arima C, Huang QM, Tomida S, Takeuchi T, Sugiyama R, Itoh Y, Yatabe Y, Goto H: **Proteasomal non-catalytic subunit PSMD2 as a potential therapeutic target in association with various clinicopathologic features in lung adenocarcinomas**. *Molecular Carcinogenesis* 2011, **50**(4):301-309.

18. Takeuchi T, Tomida S, Yatabe Y, Kosaka T, Osada H, Yanagisawa K, Mitsudomi T, Takahashi T: **Expression profile-defined classification of lung adenocarcinoma shows close relationship with underlying major genetic changes and clinicopathologic behaviors**. *Journal of clinical oncology : official journal of the American Society of Clinical Oncology* 2006, **24**(11):1679-1688.

19. Mitra R, Lee J, Jo J, Milani M, McClintick JN, Edenberg HJ, Kesler KA, Rieger KM, Badve S, Cummings OW *et al*: **Prediction of postoperative recurrence-free survival in non-small cell lung cancer by using an internationally validated gene expression model**. *Clin Cancer Res* 2011, **17**(9):2934-2946.

20. Parmigiani G, Garrett-Mayer ES, Anbazhagan R, Gabrielson E: **A cross-study comparison of gene expression studies for the molecular classification of lung cancer**. *Clin Cancer Res* 2004, **10**(9):2922-2927.

21. Raponi M, Zhang Y, Yu J, Chen G, Lee G, Taylor JM, Macdonald J, Thomas D, Moskaluk C, Wang Y *et al*: **Gene expression signatures for predicting prognosis of squamous cell and adenocarcinomas of the lung**. *Cancer Res* 2006, **66**(15):7466-7472.

22. Roepman P, Jassem J, Smit EF, Muley T, Niklinski J, van de Velde T, Witteveen AT, Rzyman W, Floore A, Burgers S *et al*: **An immune response enriched 72-gene prognostic profile for early-stage non-small-cell lung cancer**. *Clin Cancer Res* 2009, **15**(1):284-290.

23. Shedden K, Taylor JM, Enkemann SA, Tsao MS, Yeatman TJ, Gerald WL, Eschrich S, Jurisica I, Giordano TJ, Misek DE *et al*: **Gene expression-based survival prediction in lung adenocarcinoma: a multi-site, blinded validation study**. *Nat Med* 2008, **14**(8):822-827.

24. Sun Z, Wigle DA, Yang P: **Non-overlapping and non-cell-type-specific gene expression signatures predict lung cancer survival**. *J Clin Oncol* 2008, **26**(6):877-883.

25. Tang H, Xiao G, Behrens C, Schiller J, Allen J, Chow CW, Suraokar M, Corvalan A, Mao J, White MA *et al*: **A 12-gene set predicts survival benefits from adjuvant chemotherapy in non-small cell lung cancer patients**. *Clinical cancer research : an official journal of the American Association for Cancer Research* 2013, **19**(6):1577-1586.

26. Tomida S, Koshikawa K, Yatabe Y, Harano T, Ogura N, Mitsudomi T, Some M, Yanagisawa K, Takahashi T, Osada H: **Gene expression-based, individualized outcome prediction for surgically treated lung cancer patients**. *Oncogene* 2004, **23**(31):5360-5370.

27. Tomida S, Takeuchi T, Shimada Y, Arima C, Matsuo K, Mitsudomi T, Yatabe Y, Takahashi T: **Relapse-related molecular signature in lung adenocarcinomas identifies patients with dismal prognosis**. *J Clin Oncol* 2009, **27**(17):2793-2799.

28. Xie Y, Xiao G, Coombes KR, Behrens C, Solis LM, Raso G, Girard L, Erickson HS, Roth J, Heymach JV *et al*: **Robust gene expression signature from formalin-fixed paraffin-embedded samples predicts prognosis of non-small-cell lung cancer patients**. *Clin Cancer Res* 2011, **17**(17):5705-5714.

29. Zhu CQ, Ding K, Strumpf D, Weir BA, Meyerson M, Pennell N, Thomas RK, Naoki K, Ladd-Acosta C, Liu N *et al*: **Prognostic and predictive gene signature for adjuvant chemotherapy in resected non-small-cell lung cancer**. *J Clin Oncol* 2010, **28**(29):4417-4424.

30. Botling J, Edlund K, Lohr M, Hellwig B, Holmberg L, Lambe M, Berglund A, Ekman S, Bergqvist M, Ponten F *et al*: **Biomarker discovery in non-small cell lung cancer: integrating gene expression profiling, meta-analysis, and tissue microarray validation**. *Clin Cancer Res* 2013, **19**(1):194-204.

31. Der SD, Sykes J, Pintilie M, Zhu CQ, Strumpf D, Liu N, Jurisica I, Shepherd FA, Tsao MS: **Validation of a histology-independent prognostic gene signature for early-stage, non-small-cell lung cancer including stage IA patients**. *J Thorac Oncol* 2014, **9**(1):59-64.

32. Director's Challenge Consortium for the Molecular Classification of Lung A, Shedden K, Taylor JM, Enkemann SA, Tsao MS, Yeatman TJ, Gerald WL, Eschrich S, Jurisica I, Giordano TJ *et al*: **Gene expression-based survival prediction in lung adenocarcinoma: a multi-site, blinded validation study**. *Nat Med* 2008, **14**(8):822-827.

33. Hou J, Aerts J, den Hamer B, van Ijcken W, den Bakker M, Riegman P, van der Leest C, van der Spek P, Foekens JA, Hoogsteden HC *et al*: **Gene expression-based classification of non-small cell lung carcinomas and survival prediction**. *PLoS One* 2010, **5**(4):e10312.

34. Yamauchi M, Yamaguchi R, Nakata A, Kohno T, Nagasaki M, Shimamura T, Imoto S, Saito A, Ueno K, Hatanaka Y *et al*: **Epidermal growth factor receptor tyrosine kinase defines critical prognostic genes of stage I lung adenocarcinoma**. *PLoS One* 2012, **7**(9):e43923.

35. Cancer Genome Atlas Research N: **Comprehensive molecular profiling of lung adenocarcinoma**. *Nature* 2014, **511**(7511):543-550.
